# Comparison of Perioperative Outcomes of Total Laparoscopic and Robotically Assisted Hysterectomy for Benign Pathology during Introduction of a Robotic Program

**DOI:** 10.1155/2011/683703

**Published:** 2011-10-05

**Authors:** Gokhan Sami Kilic, Gradie Moore, Ayman Elbatanony, Carmen Radecki, John Y. Phelps, Mostafa A. Borahay

**Affiliations:** Department of Obstetrics & Gynecology, The University of Texas Medical Branch, Galveston, TX 77555-0587, USA

## Abstract

*Study Objective*. Prospectively compare outcomes of robotically assisted and laparoscopic hysterectomy in the process of implementing a new robotic program. *Design*. Prospectively comparative observational nonrandomized study. *Design Classification*. II-1. *Setting*. Tertiary caregiver university hospital. *Patients*. Data collected consecutively 24 months, 34 patients underwent laparoscopic hysterectomy, 25 patients underwent robotic hysterectomy, and 11 patients underwent vaginal hysterectomy at our institution. *Interventions*. Outcomes of robotically assisted, laparoscopic, and vaginal complex hysterectomies performed by a single surgeon for noncancerous indications. *Measurements and Main Results*. Operative times were 208.3 ± 59.01 minutes for laparoscopic, 286.2 ± 82.87 minutes for robotic, and 163.5 ± 61.89 minutes for vaginal (*P* < .0001). Estimated blood loss for patients undergoing laparoscopic surgery was 242.7 ± 211.37 cc, 137.4 ± 107.50 cc for robotic surgery, and 243.2 ± 127.52 cc for vaginal surgery (*P* = 0.05). The mean length of stay ranged from 1.8 to 2.3 days for the 3 methods. Association was significant for uterine weight (*P* = 0.0043) among surgery methods. *Conclusion*. Robotically assisted hysterectomy is feasible with low morbidity, a shorter hospital stay, and less blood loss. This suggests that robotic assistance facilitates a minimally invasive approach for patients with larger uterine size even during implementing a new robotic program.

## 1. Introduction

Hysterectomy is the second most commonly performed surgical procedure in the United States after cesarean delivery [1]. The Nationwide Inpatient Sample of the Healthcare Cost and Utilization Project reported 518,828 hysterectomies for benign disease in 2005 [2]. A stable rate of 5.1–5.8 hysterectomies per 1,000 female civilian US residents was reported between 1995 and 1999 [3]. The same recent analysis of Healthcare Cost and Utilization Project data showed that abdominal hysterectomy was performed in 64% of cases, followed by the vaginal route in 22% of cases and the laparoscopic route in 14%. Robotic-assisted hysterectomy is emerging as a new technique for hysterectomy [2]. 

Improved visualization and dexterity in robotic surgery may offer some advantages over conventional laparoscopy [4, 5], and shorter hospital stays and decreased blood loss may also be advantages over laparotomy [6, 7]. Overall, minimally invasive surgical techniques for performing hysterectomy have been shown to reduce patient morbidity and shorten hospital stays [8, 9]. A robotic system (da Vinci Surgical System, Intuitive Surgical, Inc., Sunnyvale, CA) is designed to address many of the limitations of conventional laparoscopy, and these benefits may allow for a minimally invasive approach in more complex cases, demonstrating the feasibility and safety of this technology as effective without increasing morbidity. However, while there have been several pilot studies on alternatives to laparoscopic hysterectomy, to date there have only been 3 larger-scale studies of robotic-assisted total hysterectomy specifically for benign indications [10–12]. In addition, limited data suggest possible advantages of robotic hysterectomy over the laparoscopic approach in complex cases, where the use of conventional laparoscopy is traditionally contraindicated [5, 13]. For example, one study reported success in treating 152 patients with complex benign pathology using robotic-assisted total hysterectomy with good patient outcomes [12]. Another study comparing laparoscopic and robotic myomectomy supported the same conclusion [14]. 

This prospective study will focus on the intra- and perioperative outcomes of robotically assisted hysterectomy for benign cases in comparison with total laparoscopic and vaginal hysterectomy. As the only tertiary teaching institution in the area, the benign cases that we see are often complex ones. In this study, we sought to investigate whether robotic assistance facilitates the surgery and results in low morbidity with acceptable perioperative outcomes.

## 2. Materials and Methods

This prospective study was conducted based on preoperative and perioperative characteristics for 3 operative types. From June 2008 to December 2009, 34 patients underwent laparoscopic hysterectomy (Group A), 25 patients underwent robotic hysterectomy (Group B), and 11 patients underwent vaginal hysterectomy (Group C) at our institution. All of these hysterectomies were performed for noncancerous indications, including adnexal mass, endometriosis, abnormal uterine bleeding, hyperplasia, dysplasia, leiomyomas, chronic pelvic pain, adenomyosis, and pelvic prolapse, among others.

To eliminate surgeon bias in our study, all surgeries were either performed by or supervised by the same surgeon. All laparoscopic and vaginal hysterectomy cases were performed by residents under the direct supervision of the first author, with more than 50% of cases performed by residents as first surgeons. In contrast, 40% of robotic cases were teaching cases. In robotic cases, resident participation was primarily in uterine manipulator placement, trocar placement, and vaginal cuff closure.

For our study, hysterectomy with or without bilateral salpingo-oophorectomy was considered to be “type 1.” Hysterectomies performed at the same time as additional surgeries were labeled as “type 2 or more.” According to this classification, 91% of laparoscopic cases were type 1 versus 84% of robotic hysterectomy cases. Of vaginal hysterectomy cases, 72.73% required 2 or more additional surgeries. 

Each case was evaluated for its complexity based on preoperative diagnosis, prior pelvic or abdominal surgery, patient's body mass index (BMI), and uterine weight. Prior pelvic or abdominal surgery was categorized as none, 1 previous surgery, or 2 or more previous surgeries. Patient BMI was categorized as BMI less than 30 or BMI of 30 or greater. Uterine weight was categorized as either <250 g or ≥250 g. All cases were further categorized as being teaching or nonteaching cases.

Before the initiation of this prospective study, institutional review board approval was obtained from The University of Texas Medical Branch at Galveston for data collection on patients who consented for surgery. All the cases were performed by a single surgeon with 7 years of experience in vaginal and laparoscopic hysterectomy, while the robotic cases were the first 25 cases in his medical career. Residents were involved in all laparoscopic and vaginal hysterectomy cases, performing more than 50% of any given case with supervision. Robotic cases were considered to be teaching cases, and resident involvement included only vaginal cuff closing. 

Patients with a uterine size not greater than 14 weeks requiring pelvic prolapse surgeries or Grade 2 Baden-Walker uterine descensus in addition to their hysterectomies were selected for the vaginal hysterectomy group. After the decision of minimally invasive hysterectomy was made, the choice between laparoscopy and robotic approach was left solely to the patient's discretion. All cases were performed under general endotracheal anesthesia. Antibiotics were given just before surgery. Patients were placed in the dorsal lithotomy position with Allen stirrups (Allen Medical Systems, Acton MA) for lower extremity positioning.

### 2.1. Robotic Hysterectomy

A Rumi System uterine manipulator with balloon tip (Cooper Surgical) was placed after appropriate preparation and draping. Patients were placed in a steep Trendelenburg position, with arms tucked at the sides and shoulder blocks placed to limit shift on the operating room table. Placement of an oral gastric tube at the beginning of the case was assured to prevent trauma to the stomach. All cases with previous abdominopelvic surgeries were started using a Veress needle to the left upper quadrant for insufflation of CO_2_ and followed by a 12-mm bladeless trocar introduced from the same location under direct visualization. In cases of no previous abdominopelvic surgeries, the Veress needle was placed in the umbilicus to obtain the necessary insufflation of CO_2_, and this was followed by introducing a 12-mm bladeless trocar for the camera at the umbilicus. Two additional 8-mm robotic trocars were placed on the patients' abdomens where additional retraction was necessary. The left upper quadrant 12-mm trocar was used on this group as well to allow the bedside surgical assistant to use a grasper or suction irrigation device. The robotic system was then docked at a 45° angle from the patient's left foot side. All cases were performed using monopolar EndoWrist scissors (Intuitive Surgical, Inc.) combined with a PK grasper. Hysterectomies were classified as American Association of Gynecologic Laparoscopists type IVE, defined as total laparoscopic removal of the uterus and cervix including vaginal cuff closure, and performed using the KOH Colpotomizer System (Cooper Surgical). 

All pathologic specimens were removed using 1 of the 3 following methods: direct removal through the vaginal cuff opening, morcellation of the specimen using an endoscopic morcellator, or sectioning of the uterine specimen with the robot using an EndoWrist monopolar cautery instrument (Institutive Surgical, Inc.) to create portions small enough to be delivered vaginally. The vaginal cuff was closed robotically using 0 polyglactin (Vicryl, Ethicon Endo-Surgery, Inc., Cincinnati, OH) on a CT-1 needle in a single separate suture closure. 

Sutures were instrument-tied using the robotic instruments. Patients were desufflated, and pedicles were checked at half desufflation for homeostasis. The robotic system was then undocked, and all trocars were removed under direct visualization. The 12-mm trocar sites received a single deep 0-polyglactin suture, and all skin incisions were closed with 4-0 polyglactin subcuticular sutures. Adhesive skin closures (Steri-Strips, 3M, St. Paul, MN) were placed as dressing. 

The following times were recorded: docking time, defined as the time from first incision to placement of the robotic instruments into the patient, morcellation time, and total operative time, defined as time from when the patient was brought into the OR until she left the room. In addition, uterine weight, blood loss, conversions, and intraoperative and postoperative complications requiring intervention, as well as length of hospital stay, were monitored and recorded. Cases were stratified based on the level of complexity using BMI, uterine weight, presence of prior pelvic or abdominal surgeries, and preoperative diagnosis. We also reported outcomes for subgroups of patients with uterine weight of ≥250 g or BMI ≥ 30.

### 2.2. Laparoscopic Hysterectomy

Laparoscopic hysterectomy cases were done with the same trocar placement technique and locations applied in robotic cases, but, instead of an 8-mm robotic port, 25-mm bladeless trocars were used. Other than docking time, the same parameters were recorded as for robotic cases.

### 2.3. Vaginal Hysterectomy

The cervix was grasped with a tenaculum, and the cervicovaginal junction was circumferentially injected with a vasoconstrictive agent. A circumferential incision was made at the cervicovaginal junction, and the vagina was dissected off the cervix for several centimeters. Careful entry into the anterior and posterior cul-de-sacs was done using sharp dissection. Retraction of the bladder anteriorly off the cervix, visualization of the lower edge of the peritoneum, and careful sharp entry into the anterior cul-de-sac minimized the chance of cystotomy. After the peritoneum was entered anteriorly and posteriorly, the uterosacral and cardinal ligament complex was detached from the uterus bilaterally. Downward traction on the cervix brought the uterus and its remaining attachments closer to the operator, where they too could be seen, clamped, and transected under direct visualization. 

A McCall's culdoplasty or simple peritoneal closure and intraperitoneal uterosacral ligament plication were performed after a vaginal hysterectomy to prevent vaginal vault prolapse. The vaginal cuff was closed by using 0-polyglactin CT-1 needle in a running suture closure.

### 2.4. Data Analysis

Patients' preoperative and operative characteristics were summarized using means, standard deviations and 95% confidence interval for the continuous variables, and proportions for discrete variables among the 3 surgery groups (laparoscopic, robotic, and vaginal). The 1-way analysis of variance (ANOVA) was used to compare the means of continuous variables among the surgery groups. The chi-square test was used to investigate the association between category variable and the surgery groups, and the normality of continuous variables was tested. The tests were assessed at the 0.05 level of significance. Statistical computations were carried out using statistical software, SAS release 9.2.

## 3. Results

A total of 70 consecutive patients underwent hysterectomy with laparoscopic, robotic, and vaginal techniques for benign indications in 12 months. Patient characteristics and surgical indications are listed in Table 1. The average age for the patients was 42.5 ± 8.78 years for laparoscopic hysterectomy, 46.6 ± 7.54 years for robotic hysterectomy, and 44.1 ± 10.72 for vaginal hysterectomy. Overall, 58.82% of patients had a BMI equal to or exceeding 30, and percentages for robotic and vaginal hysterectomy were 64.0% and 36.36%, respectively. Cervical dysplasia and leiomyomas were the main indication (67.6%) for laparoscopic hysterectomy patients. Adenomyosis and leiomyomas were the main indications (84%) for robotic hysterectomy patients. Pelvic prolapse and SUI were the only reasons for vaginal hysterectomy (100%). The racial composition of patients included 61.76% white, 20.59% African American, and 17.65% Hispanic for laparoscopic hysterectomy; 40% white, 48% African American, and 12% Hispanic for robotic hysterectomy; 72.73% white, 18.18% African American, and 9.09% Hispanic for vaginal hysterectomy. The majority of laparoscopic hysterectomy patients (76%) had a history of prior abdominal/pelvic surgery. In the same group, 35.29% had undergone 2 or more prior abdominal/pelvic surgeries. Among robotic hysterectomy patients, 76% had a history of prior abdominal/pelvic surgery. Of these women, 32% had undergone multiple prior abdominal/pelvic surgeries. For vaginal hysterectomy patients, more than 45% had undergone prior abdominal/pelvic surgery. Only 18.18% had multiple previous abdominal/pelvic surgeries. Regarding uterine weight, 14.71% of patients in the laparoscopy group had a uterine weight greater than 250 g versus 44% for the robotic group. All vaginal hysterectomy cases revealed a uterine weight less than 250 g. 

Total operative time was, on average, 216.3 minutes, which included morcellation time when applicable for laparoscopic patients (Table 2), 298.8 minutes for robotic patients, and 163.5 minutes for vaginal hysterectomy. When morcellation of the uterus was taken out of the operative time, the mean values were as follows: 208.3 minutes for laparoscopic, 286.2 minutes for robotic, and 163.5 minutes for vaginal cases. Room time was documented independently of operative time, and the mean values were 281.4 minutes for laparoscopic, 378.1 minutes for robotic, and 218.5 minutes for vaginal cases. Uniquely, the docking time was only applicable for robotic cases, and its mean value was 21.5 minutes. Average blood loss for laparoscopic hysterectomy patients in this study was calculated to be 242.7 mL (50 mL–1000 mL), compared to robotic hysterectomy patients with average blood loss of 137.4 mL (20 mL–400 mL) and 243.2 mL (50 mL–500 mL) for vaginal hysterectomy patients. 

Laparoscopic patients had an average length of stay of 2.3 ± 1.06 days (range 1–5 days). Robotic patients had an average length of stay of 1.8 ± 0.88 days (range 1–4 days). Vaginal hysterectomy patients had an average length of stay of 2.00 ± 1.00 days (range 1–4 days).

In the robotic and laparoscopic groups, there was 1 serious intraoperative complication in each group. This included 1 ureteral injury and 1 bowel injury. Also, there were 4 cases in the laparoscopic group and 3 in the robotic group who had mild intraoperative complications. These included uterine artery bleeding, vaginal laceration, IV infiltration, incorrect count, serosal bowel injury, and serosal bladder injury. There was no complication in the vaginal hysterectomy cases. Among the laparoscopic cases, the uterine artery bleeding and bowel injury cases were converted to minilaparotomy. Among the robotic cases, 1 case with severe adhesions led to a ureteral injury that was corrected by minilaparotomy. Another case was converted to open due to intolerance of the Trendelenburg position. Two patients from each of the robotic and laparoscopic groups received 2 units of packed RBCs intraoperatively. Among these 4 patients, only 1 required blood due to intraoperative excessive bleeding while the remaining 3 were due to severe preoperative anemia, which was treated by transfusion at the time of surgery. 

There were 10 laparoscopic hysterectomy patients with postoperative complications, including urinary retention, irregular blood pressure, deep venous thrombosis, trocar site pain, cuff cellulitis, and urinary tract infection. Among the laparoscopic cases, 1 patient had both cuff cellulitis and anemia, while the remaining 9 cases comprised the rest of the previous complications. In the robotic cases, 5 patients experienced postoperative complications. One of the robotic patients had both cuff cellulitis and trocar site pain. The remaining 4 patients had complications, such as irregular blood pressure, urinary tract infection, and trocar site pain. Among the vaginal hysterectomy cases, 3 patients had postoperative complications. Two of them had urinary tract infections, and 1 had urinary retention. None of our postoperative patients required blood transfusion, and no patient needed to go to the operative room for a second time related to the initial surgery.

The percentage of cases with intraoperative challenges was 35.29% for laparoscopic, 56% for robotic, and 0% for vaginal procedures. Intraoperative challenges included dense pelviabdominal adhesions, lower uterine segment fibroids extending to the uterine artery, morcellated cases, adnexal masses requiring lateral pelvic wall dissection, an inability to obtain an appropriate Trendelenburg position, and malfunction of the instruments. 

A multiple regression model was used to determine the factors influencing operative time. Prior pelvic surgery, classification as a teaching case, and BMI did not affect operative time. The variables that were significantly associated with increased operative time were inclusion in the laparoscopic or robotic groups (Groups A and B), type of surgery, and estimated blood loss (Table 3).

## 4. Discussion

This prospective study presents our initial experience with robotic-assisted total hysterectomies for benign indications in 25 consecutive cases compared to 34 laparoscopic and 11 vaginal hysterectomies, also for benign indications. The inclusion criteria for vaginal hysterectomies were Baden-Walker Grade 2 or higher symptomatic uterine, bladder, or rectum prolapse; required additional vaginal surgeries; and a uterus not greater than 14 weeks in size. Due to this relative selection bias, we could not randomize the patients among the 3 groups. However, the same criteria were used for certain perioperative outcomes, such as recovery time. To prevent surgeon selection bias, the decision to have robotic versus laparoscopic surgery was left to the patients after risks, benefits, and exact procedures were explained in detail. 

Robotic gynecologic procedures were FDA approved in March 2005 [1]. Published average times for robotic hysterectomies vary from 122.9 minutes to 242 minutes in mainstream journals [10, 11, 15–17]. The operative time in our study, 286.2 minutes—slightly longer than average—was affected by multiple factors. In our study, operative time was reported from “skin to skin,” which is defined as from when the surgeon started to perform the vaginal exam under general anesthesia until the last suture was placed to close the trocar sites, instead of starting from uterine manipulator or skin incision. The single interrupted suture technique used to close the vaginal cuff versus the continuous running suture may have an impact on operating time. Additionally, technical intraoperative problems required changing the camera, cord, and light source, and a visualization problem in the robotic system was included in our operative time. 

The robotic cases, reported to explore the learning curve, were the first 25 performed by the surgeon, residents, and the OR team. One observation about the learning curve is that the first 15 cases averaged 308.5 minutes, while the next 10 cases averaged only 252.8 minutes. Room time, used as an indicator of ancillary team efficiency, dropped from 407.8 minutes in the first 15 cases to 333.6 minutes in the following 10 cases. Our data revealed that after the first 15 cases of robotic surgery, operating time was not statistically different from our laparoscopic cases (*P* = 0.1179). Current data suggests the learning curve to be between 20 and 100 cases, [11, 15, 18] and our study supports the lower side of the variation.

Our experience indicates that initiating a new robotic program requires more team training than any other procedures. This includes technical personnel, scrub nurses, and circulating nurses available in the operating room. The team should be doubled to be able to substitute when needed. Interdepartmental collaboration in the related specialties for combined surgeries and intraoperative consultation needs to be developed simultaneously. Administrative support is also essential to create robotic programs. These are all independent factors that affect operative time and quality of care in robotic surgeries.

In laparoscopy and robotic cases, BMIs higher than 30 were predominant (58% and 64%, resp.), in contrast to vaginal hysterectomies (36%). Uterine weight more than 250 g was more common in the robotic group (44%). Estimated blood loss was least in robotic cases, with 137 mL. Previous abdominopelvic surgery was more common in robotic and laparoscopic cases compared to vaginal cases (76% and 76.4%, resp., versus 45.4%). Length of hospital stay was least with robotic cases (robotics 1.8 days, vaginal 2.0 days, and laparoscopic 2.3 days). While recognizing that our study included only a limited number of cases, we found that robotic hysterectomy seems to be a good approach for patients with high BMI, a larger uterus, or a history of previous abdominopelvic surgeries. Robotic surgery provides a safe and fast recovery as well. 

Robotic hysterectomy has only recently emerged in the gynecology field. Gynecologists need more scientific data to address specific indications, advantages, and disadvantages of different surgical approaches during the informed consent process. Our study is the first to compare robotic, laparoscopic, and vaginal hysterectomies using multiple perioperative criteria performed by a single surgeon. We could not overcome the selection bias in some of our patients on the vaginal approach since additional vaginal surgeries were required. However, our preoperative characteristics other than uterine weight were not statistically different among the 3 groups. Surgery time was shorter in vaginal hysterectomies, even with the additional pelvic surgeries included, compared to laparoscopic cases. These results agree with Falcone, whose review compared laparoscopic, vaginal surgeries, and abdominal hysterectomies. Minimally invasive laparoscopic hysterectomy not only had a longer operation time but also had faster recovery, shorter hospital stays, and lower EBL compared to the abdominal method [1]. Based on these criteria, it is clear to see the superiority of laparoscopic and vaginal hysterectomies over the abdominal approach. Vaginal hysterectomy should be the first choice in selected patients, especially when cost effectiveness is brought into the equation. However, minimally invasive surgeries are increasing their presence nationwide, and there are strong indications to predict that the trend will continue. Laparoscopic versus robotic approaches still require more exploration to identify the patient's characteristics to offer the more suitable surgery between these 2 hysterectomy types.

## Figures and Tables

**Table 1:** Preoperative characteristics.

	Group A, laparoscopic (*n* = 34)	Group B, robotic (*n* = 25)	Group C, vaginal (*n* = 11)	*P*
Age				0.2101
*N*	34	25	11	
Mean (SD)	42.5 (8.78)	46.6 (7.54)	44.1 (10.72)	
95% CI	(39.4, 45.5)	(43.5, 49.7)	(36.9, 51.3)	
Range	(27, 60)	(30, 64)	(32, 63)	

Body mass index				0.2925
More than 30	20 (58.82%)	16 (64.00%)	4 (36.36%)	
Less than 30	14 (41.18%)	9 (36.00%)	7 (63.64%)	

Uterine weight (g)				0.0043
Less than 250 g	29 (85.29%)	14 (56.00%)	11 (100%)	
More than 250 g	5 (14.71%)	11 (44.00%)		

Race				0.1534
African American	7 (20.59%)	12 (48.00%)	2 (18.18%)	
White	21 (61.76%)	10 (40.00%)	8 (72.73%)	
Hispanic	6 (17.65%)	3 (12.00%)	1 (9.09%)	

Prior pelvic or abdominal surgery				0.3523
None	8 (23.53%)	6 (24.00%)	6 (54.55%)	
1 occurrence	14 (41.18%)	11 (44.00%)	3 (27.27%)	
2 and more occurrences	12 (35.29%)	8 (32.00%)	2 (18.18%)	

Type of surgery				<.0001
1	31 (91.18%)	21 (84.00%)	3 (27.27%)	
2 and more	3 (8.82%)	4 (16.00%)	8 (72.73%)	

Indication for surgery				
Adnexal mass	3	7	0	
Endometriosis	2	1	0	
Abnormal uterine bleeding	9	3	0	
Hyperplasia	1	0	0	
Dysplasia	13	1	3	
Leiomyomas	10	11	0	
SUI	2	2	4	
Chronic pelvic pain	3	1	0	
Adenomyosis	2	10	1	
Pelvic prolapse	0	0	7	
Other	2	6	3	

**Table 2:** Operative characteristics.

	Group A, laparoscopic (*n* = 34)	Group B, robotic (*n* = 25)	Group C, vaginal (*n* = 11)	*P*
Docking time (min)				
*N*		25		
Mean (SD)		21.5 (10.48)		
95% CI		(17.2, 25.9)		
Range		(4, 50)		

Room time (min)				<.0001
*N*	34	25	11	
Mean (SD)	281.4 (67.01)	378.1 (91.45)	218.5 (61.86)	
95% CI	(258.0, 304.8)	(340.4, 415.9)	(176.9, 260.0)	
Range	(172, 504)	(207, 529)	(117, 310)	

Operative time (min)				<.0001
*N*	34	25	11	
Mean (SD)	216.3 (62.74)	298.8 (89.70)	163.5 (61.89)	
95% CI	(194.4, 238.2)	(261.8, 335.9)	(121.9, 205.0)	
Range	(132, 452)	(127, 466)	(70, 254)	

Operative time not including morcellation (min)				<.0001
*N*	34	25	11	
Mean (SD)	208.3 (59.01)	286.2 (82.87)	163.5 (61.89)	
95% CI	(187.7, 228.9)	(252.0, 320.5)	(121.9, 205.0)	
Range	(132, 452)	(127, 466)	(70, 254)	

Type of cases				
Teaching cases	34 (100%)	10 (40.00%)	11 (100%)	<.0001
Nonteaching cases		15 (60.00%)		

Estimated blood loss (cc)				0.0505
*N*	34	25	11	
Mean (SD)	242.7 (211.37)	137.4 (107.50)	243.2 (127.52)	
95% CI	(168.9, 316.4)	(93.0, 181.8)	(157.5, 328.9)	
Range	(50, 1000)	(20, 400)	(50, 500)	

Length of stay (days)				0.1280
*N*	34	25	11	
Mean (SD)	2.3 (1.06)	1.8 (0.88)	2.0 (1.00)	
95% CI	(1.9, 2.7)	(1.4, 2.1)	(1.3, 2.7)	
Range	(1, 5)	(1, 4)	(1, 4)	

Conversions				0.8317
Yes	2 (5.88%)	2 (8.00%)		
No	32 (94.12%)	23 (92.00%)	11 (100%)	

Intraoperative complications				0.8610
0	29 (85.29%)	20 (83.33%)	11 (100%)	
1	4 (11.76%)	3 (12.50%)		
2	1 (2.94%)	1 (4.17%)		

Postoperative complications				0.8883
0	24 (85.29%)	20 (80.00%)	8 (72.73%)	
1	9 (26.47%)	4 (16.00%)	3 (27.27%)	
2	1 (2.94%)	1 (4.00%)		

Challenges				0.0056
Yes	12 (35.29%)	14 (56.00%)		
No	22 (64.71%)	11 (44.00%)	11 (100%)	

**Table 3:** Regression model: the effects of preoperative characteristics on operative time.

	Parameter estimate	Standard error	*t*	*P*
Intercept	58.68	42.98	1.37	0.1771
Group (A)	107.63	31.76	3.39	0.0012
Group (B)	170.72	35.46	4.81	<.0001
Prior pelvic surgery	−10.44	7.85	−1.33	0.1882
Teaching case (NT)	48.91	29.45	1.66	0.1018
Type of surgery	38.17	15.12	2.52	0.0142
Body mass index (less than 30)	−17.78	17.37	−1.02	0.3102
Estimated blood loss	0.12	0.05	2.42	0.0183
